# HIF-1α increases the osteogenic capacity of ADSCs by coupling angiogenesis and osteogenesis via the HIF-1α/VEGF/AKT/mTOR signaling pathway

**DOI:** 10.1186/s12951-023-02020-z

**Published:** 2023-08-07

**Authors:** Shuang Song, Guanhua Zhang, Xutao Chen, Jian Zheng, Xiangdong Liu, Yiqing Wang, Zijun Chen, Yuxi Wang, Yingliang Song, Qin Zhou

**Affiliations:** 1 Key Laboratory of Shaanxi Province for Craniofacial Precision Medicine Research, College of Stomatology, Xi’an Jiaotong University, Xi’an, 710004 China; 2 Department of Oral Implants, School of Stomatology, State Key Laboratory of Military Stomatology and National Clinical Research Center for Oral Diseases and Shaanxi Engineering Research Center for Dental Materials and Advanced Manufacture, The Fourth Military Medical University, Xi’an, 710032 China; 3 Department of Prosthodontics, Peking University School and Hospital of Stomatology, Beijing, 100081 China

**Keywords:** Hypoxia-inducible factor 1α, Adipose-derived stem cells, Osteointegration, Osteogenic differentiation, Stem cell transplantation, HIF-1α/VEGF/AKT/mTOR signaling pathway, Titanium implant

## Abstract

**Background:**

Stabilization and increased activity of hypoxia-inducible factor 1-α (HIF-1α) can directly increase cancellous bone formation and play an essential role in bone modeling and remodeling. However, whether an increased HIF-1α expression in adipose-derived stem cells (ADSCs) increases osteogenic capacity and promotes bone regeneration is not known.

**Results:**

In this study, ADSCs transfected with small interfering RNA and HIF-1α overexpression plasmid were established to investigate the proliferation, migration, adhesion, and osteogenic capacity of ADSCs and the angiogenic ability of human umbilical vein endothelial cells (HUVECs). Overexpression of HIF-1α could promote the biological functions of ADSCs, and the angiogenic ability of HUVECs. Western blotting showed that the protein levels of osteogenesis-related factors were increased when HIF-1α was overexpressed. Furthermore, the influence of upregulation of HIF-1α in ADSC sheets on osseointegration was evaluated using a Sprague–Dawley (SD) rats implant model, in which the bone mass and osteoid mineralization speed were evaluated by radiological and histological analysis. The overexpression of HIF-1α in ADSCs enhanced bone remodeling and osseointegration around titanium implants. However, transfecting the small interfering RNA (siRNA) of HIF-1α in ADSCs attenuated their osteogenic and angiogenic capacity. Finally, it was confirmed in vitro that HIF-1α promotes osteogenic differentiation and the biological functions in ADSCs via the VEGF/AKT/mTOR pathway.

**Conclusions:**

This study demonstrates that HIF-1α has a critical ability to promote osteogenic differentiation in ADSCs by coupling osteogenesis and angiogenesis via the VEGF/AKT/mTOR signaling pathway, which in turn increases osteointegration and bone formation around titanium implants.

**Graphical Abstract:**

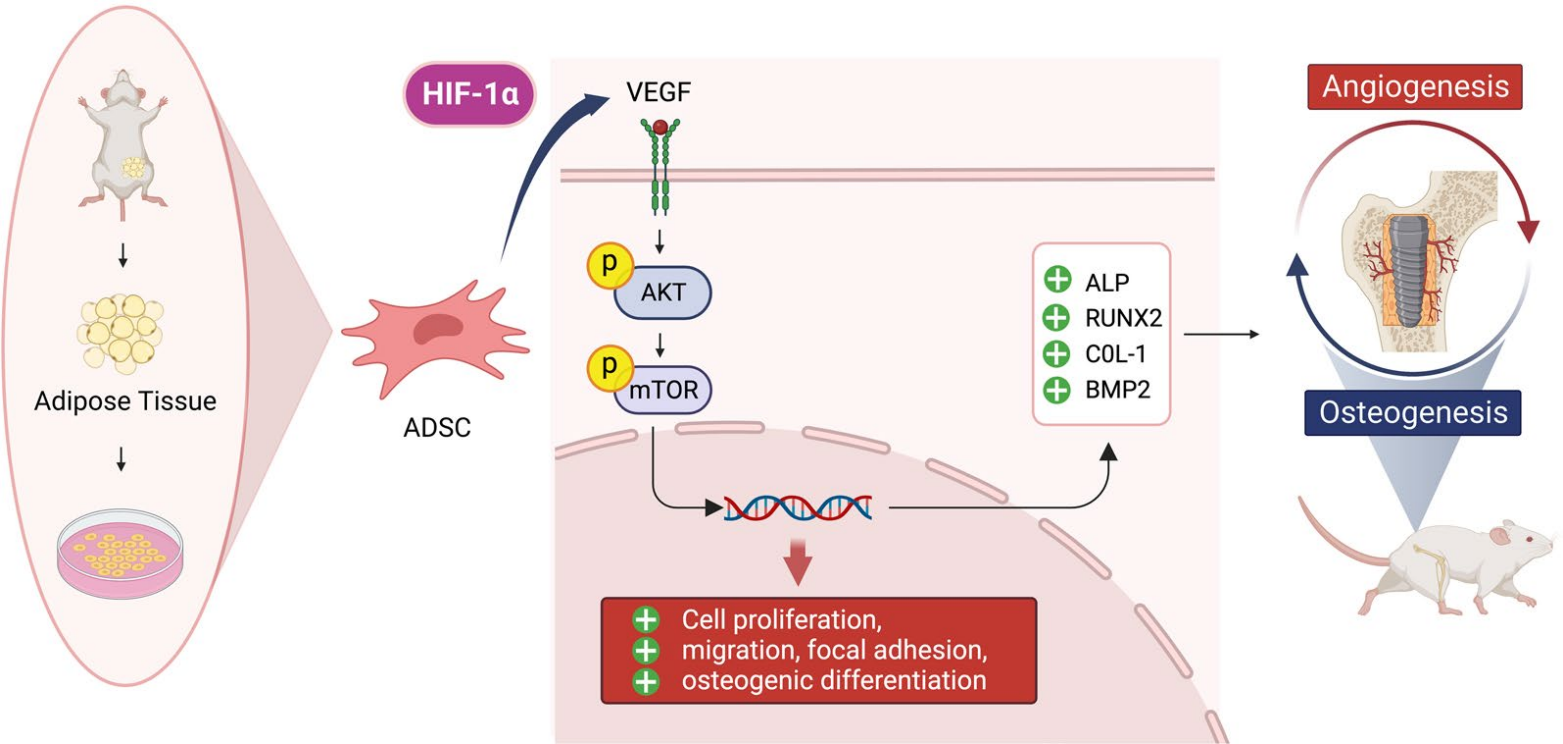

**Supplementary Information:**

The online version contains supplementary material available at 10.1186/s12951-023-02020-z.

## Background

Implant-supported prosthodontics is a standardized and predictable method has been widely used in the treatment of edentulous patients [1]. Although the 5‐year survival rates of implant-based restorations can reach more than 95% [2, 3], implant failure occurs in some cases [4], which leads to additional costs and lost time for patients. Therefore, it is very important to improve the bone-implant contact rate and implant success rate.

Bone marrow mesenchymal stem cells (BMSCs) play a vital role in bone remodeling, but their retrieval is invasive, and the numbers of stem cells that can be isolated is limited [5–7]. Adipose-derived stem cells (ADSCs), a type of mesenchymal stem cell harvested during liposuction procedures, have been found to offer significant potential for stem cell-based bone tissue engineering owing to their abundancy, self-renewal ability, and multi-potential differentiation [8–11]. The osteogenic potential of ADSCs in their repair of maxillofacial bone defects has been demonstrated in animal models and clinical trials [12, 13]. However, the inherent tendency of these cells to undergo adipocyte differentiation, with the poor engraftment and limited osteogenic differentiation, has greatly impeded the clinical application of ADSCs in bone tissue engineering [14–16]. Challenges still remain to the use of adipose-derived stem cells alone to repair bone tissues [17]. Therefore, a reliable and predictable induction of specific osteogenic differentiation of ADSCs is key in bone tissue regeneration.

Hypoxia-inducible factor 1 (HIF-1) was reported as a pivotal nuclear factor for transcriptional activation under low O_2_ concentrations (hypoxia); it acts as a cellular oxygen sensor and plays an important role in the regulation of bone homeostasis and angiogenesis [18]. HIF-1 is a basic heterodimeric helix-loop-helix protein, consisting of two subunits HIF-1α and HIF-1β. The HIF-1α subunits is degraded under conditions of sufficient O_2_ bioavailability (normoxia), but under hypoxic conditions, it remains stable [19, 20]. The expression of HIF-1α plays a vital role in preventing bone metabolic diseases under pathological conditions [21, 22]. To date, the biological function of HIF-1α in the skeletal system, especially in the bone marrow mesenchymal stem cells (BMSCs) and bone marrow-derived macrophages (BMMs), has been widely studied [21]. HIF-1α and its target genes have been identified to play roles in a multitude of biological processes, including cell survival, angiogenesis, osteoprogenitor cell recruitment, and bone formation induction [23–31]. For example, the stabilization of HIF-1α in BMSCs may directly be contribute to subsequent gene transcription, including the transcription of RUNX2 [32], which acts as a transcription factor and plays a key role in osteogenesis, as well as the transcription of OPG [33, 34], BMP2 [35], VEGF [36], PGF [37], and SDF-1 [38]. Recent studies have investigated the coupling of HIF-1α-driven bone formation with angiogenesis induced by VEGF upregulation in vivo [39], and other studies have demonstrated that the stabilization of HIF-1α markedly increased osteoblast numbers and dramatically stimulates cancellous bone formations in the osteoblast precursors [40]. Moreover, increasing HIF-1α expression was reported to improve angiogenic and osteogenic differentiation, thus prompting osteogenesis in BMSCs [41, 42]. This suggests that HIF-1α plays important roles in balancing bone homeostasis and promoting osteogenic differentiation. Whether the expression of HIF-1α also alters the biological functions and affects the osteogenic differentiation of ADSCs remains unclear.

Studies have reported that the VEGF/AKT/mTOR signaling pathway also plays an important role in angiogenesis and osteogenesis [43–45]. For example, activation of the VEGF/AKT/mTOR signaling pathway can promote angiogenesis and the differentiation of bone marrow mesenchymal stem cells [46, 47], thus increasing bone formation [48, 49]. During this process, HIF-1α, as a transcription factor, can regulate VEGF transcription and protein expression [50], thereby activating the VEGF/AKT/mTOR signaling pathway and promoting osteogenesis [51]. Whether or not HIF-1α can promote osteogenesis via the VEGF/AKT/mTOR signaling pathway in ADSCs has not yet been reported.

In the present study, we provided evidence that HIF-1α can promote angiogenesis in human umbilical vein endothelial cells (HUVECs), and positively affect the biological functions of adipose-derived mesenchymal stem cells (ADSCs), such as proliferation, migration, and osteogenic potential. In vivo, the transplantation of ADSC sheets with overexpression of HIF-1α into a Sprague–Dawley (SD) rat implantation model markedly increased osseointegration, whereas the transplantation of HIF-1α-silenced ADSC sheets reduced osteointegration. The key finding of this research is that an effective stem cell-based bone tissue engineering material should be used to improve peri-implant osteogenesis in combination with a new therapeutic strategy targeting HIF-1α to promote implant osseointegration.

## Results

### Characterization of ADSCs

After isolating ADSCs from male SD rats, we obtained passage 3 cells for our experiment, which appeared to have a fibroblast-like spindle-shaped morphology (Additional file 1: Fig. S1A). Calcified nodules were stained via Alizarin red staining after 21 days of osteogenic differentiation (Additional file 1: Fig. S1B), and oil red O-stained ADSCs contained lipid droplets after 7 days of adipogenic differentiation (Additional file 1: Fig. S1C). Flow cytometry showed that ADSCs were clearly positive for CD29, CD44, and CD105, but negative for CD45 was negative (Additional file 1: Fig. S1D).

### Silencing HIF-1α in ADSCs impairs cell proliferation, migration, and adhesion and attenuates osteogenic differentiation

To investigate the role of the HIF-1α gene in ADSCs, three siRNAs (si-HIF-1α #174, 837, and 1178) were utilized to downregulate the HIF-1α expression in ADSCs under hypoxic conditions (2% O_2_) (Additional file 1: Fig. S2). The silencing efficiency of these siRNAs was verified by qRT‒PCR, and si-HIF-1α #837 was selected as the most effective siRNA. As a result, it was chosen for further functional studies while cell proliferation, migration and adhesion ability, and osteogenic capacity were evaluated separately.

**Fig. 1:**
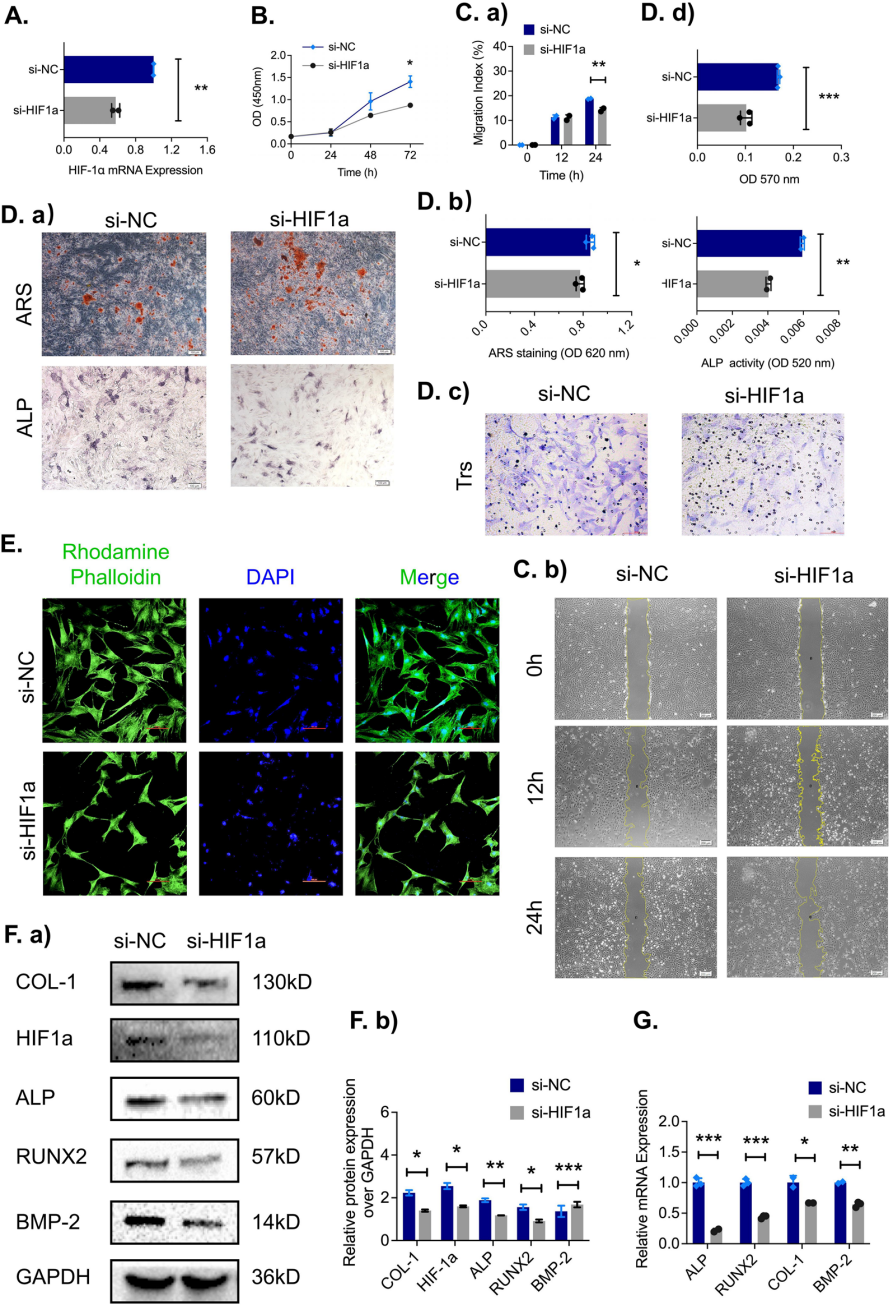
Silencing HIF-1α in ADSCs impairs cell biological functions and suppresses osteogenic capacity in vitro. **A** The transfection efficiency of si-HIF1α mRNA in ADSCs was measured by qRT‒PCR (n = 3). **B** The proliferation of ADSCs transfected with HIF-1α-siRNA and nontargeting si-NC mRNA was detected by the CCK-8 assay (n = 3). **C** a, b The migration of ADSCs transfected with si-NC and si-HIF1α was detected by the scratch wound assays at 0, 12, and 24 h (scale bar = 200 μm). After osteoblast induction, (**D**) **a**, **b** Alizarin red staining for mineralized nodules was performed after 21 days, and BCIP/NBT staining for alkaline phosphatase was performed after 7 days. **D** **c**, **d** The migration ability of transfected ADSCs by Transwell test assays at 24 h (scale bar = 100 μm). **E** Representative images of ADSC focal adhesion plaques using immunofluorescence staining (F-actin in green and nuclei in blue, scale bar = 100 μm). **F**, **G** mRNA expression levels and protein levels of osteogenic-related genes (ALP, RUNX2, COL1, and BMP2) in transfected ADSCs. *p < 0.05; **p < 0.01; ***p < *0.001*

The mRNA level of the HIF-1α gene decreased significantly after siRNA transfection (Fig. 1A), and the CCK-8 assay showed that the proliferation of ADSCs in the si-HIF1α group was markedly inhibited relative to that of the si-NC group after 72 h (Fig. 1B). In addition, compared to the negative control ADSCs, ADSCs subjected to HIF-1α silencing exhibited weaker migration ability, as quantified by a scratch wound healing assay (Fig. 1C a, b), and a Transwell assay (Fig. 1D c, d). More importantly, immunofluorescence staining in the adhesion test revealed that the total number of focal adhesion plaques in ADSCs transfected with si-HIF-1α was significantly decreased compared with that in ADSCs transfected with si-NC, and the ADSCs in the negative control group were fully stretch, flattened and well attached to the titanium plate surface (Fig. 1E).

The stages of osteogenic differentiation in ADSCs were defined by ALP and calcium deposition. After osteogenic induction, quantitative analysis of ARS and ALP staining showed that si-HIF-1α inhibited calcium deposition and osteogenic differentiation compared to the negative control in ADSCs (Fig. 1D a-b). Similar results were obtained for the protein and mRNA expression of osteogenesis associated genes. ALP, RUNX2, COL-1, and BMP-2 expression decreased significantly in the group subjected to HIF-1α group (Fig. 1F, G).

These results collectively revealed that silencing HIF-1α in ADSCs can significantly impair their proliferation, migration, and adhesion potential and may attenuate their osteogenic capacity.

### Overexpression of HIF-1α promotes proliferation, migration, and focal adhesion formation and enhances osteogenic capacity in ADSCs

**Fig. 2:**
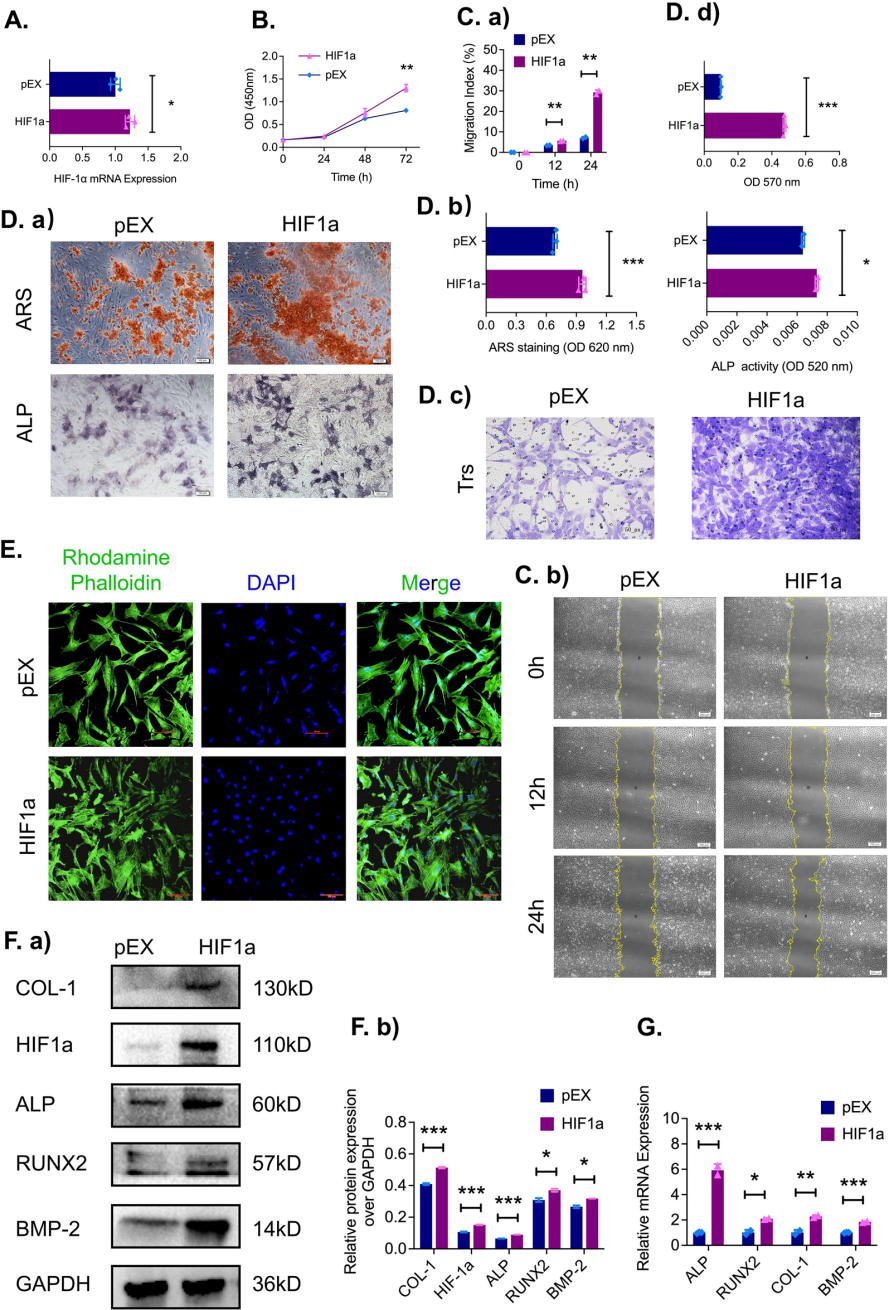
HIF-1α overexpression in ADSCs significantly increases cell proliferation and osteogenic capacity in vitro. **A** The mRNA expression of HIF-1α in ADSCs after transfection with a HIF-1α overexpression plasmid and an empty plasmid (pEX) was evaluated by qRT‒PCR (n = 3). **B** The proliferation of transfected ADSCs was evaluated by a CCK-8 assay (n = 3). **C** **a**, **b** The scratch assays at 0, 12 and 24 h (n = 3). **D** **a**, **b** ARS and BCIP/NBT staining after osteogenic induction. **D** **c**, **d** Representative images and quantitative analysis of Transwell assays after 24 h (n = 3, scale bar = 100 μm). **E** Representative images of ADSC focal adhesion plaques using immunofluorescence staining (F-actin in green and nuclei in blue, scale bar = 100 μm). **F** **a**, **b** Protein levels and (**G**) mRNA and of the osteogenic marker genes (ALP, RUNX2, COL1, and BMP2). *p < 0.05; **p < 0.01; ***p < *0.001*

We next asked whether or not the overexpression HIF-1α could enhance proliferation, migration, and adhesion and promote osteogenic differentiation in ADSCs. ADSCs were cultured until they reached 60–70% confluence, and plasmids containing HIF-1α cDNA (HIF1α) or an empty plasmid (pEX) was transfected. To evaluate the overexpression efficiency, qRT‒PCR was to be performed and revealed that HIF-1α mRNA expression levels increased significantly in the HIF-1α group relative to the negative control group (Fig. 2A).

In terms of proliferation, the CCK-8 assay showed markedly higher proliferation in the HIF-1α group than in the negative control group after 72 h (Fig. 2B). Additionally, ADSCs overexpressing HIF-1α exhibited strong migration ability, as demonstrated by quantitative analysis of the scratch (Fig. 2C, a, b) and migration assay results (Fig. 2D c, d). We labeled both F-actin (green) and nuclei (blue) in the immunofluorescence assays and the results showed that the number of attached ADSCs in the HIF-1α group was higher than that in the negative control group (Fig. 2E). The mineralization level was visualized and quantified by Alizarin red staining and ALP staining, revealing that the calcium deposition in the HIF-1α group was markedly increased compared to that in the negative control group (Fig. 2D a, b).

To further confirm these findings, ADSCs transfected with HIF-1α were subjected to qRT‒PCR analyses to evaluate osteogenic marker genes. HIF1α overexpression in ADSCs significantly increased the expression of bone-related genes, such as ALP, RUNX2, COL-1, and BMP2 (Fig. 2G). Western blotting analysis also showed that the protein levels of osteogenic markers were elevated after HIF-1α overexpression (Fig. 2F a, b).

Taken together, these results demonstrated that the proliferation, migration, focal adhesion, calcium deposition, and osteogenic differentiation ability of ADSCs were improved in the HIF-1α overexpression group. Osteogenic marker gene expression was also markedly increased.

### The ability of HIF-1α to induce angiogenesis in HUVECs

**Fig. 3:**
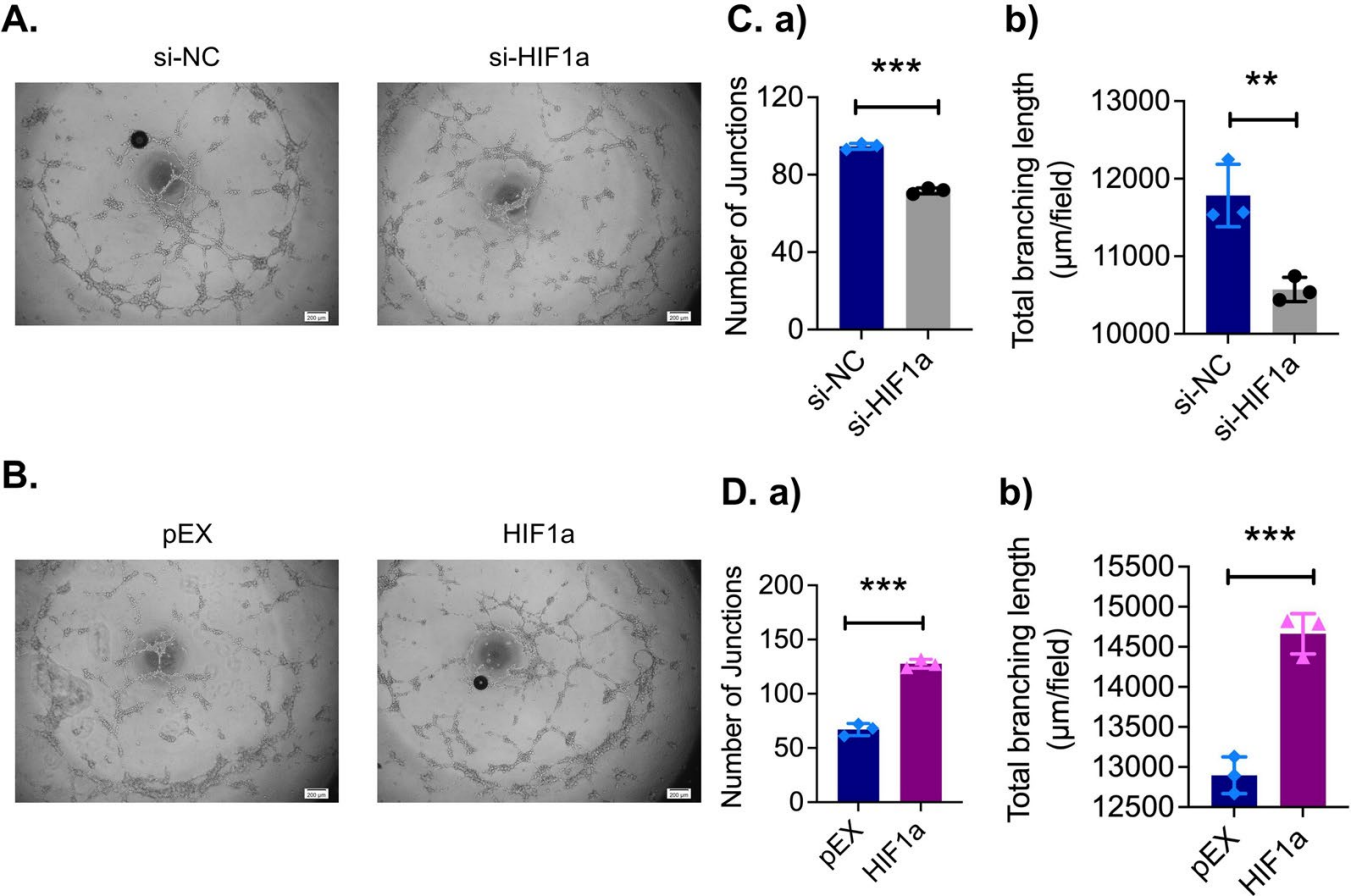
HIF-1α promoted angiogenesis in HUVECs in vitro. **A**, **B** HUVECs were subjected to an in vitro tube formation assay after transfected with a small interfering RNA targeting HIF-1α (si-HIF-1α) or a plasmid containing HIF-1α cDNA (HIF-1α) for 24 h (Scale bar = 200 μm). **C**, **D** Quantitative analysis of the data from the tube formation assay. *p < 0.05; **p < 0.01; ***p < *0.001*

The effects of the HIF-α gene on endothelial cell tube formation were further investigated in a Matrigel plug assay. Silencing of HIF-1α by siRNA resulted in significant decreases in both the number of junctions and the total branch length in comparison to those of control siRNA treated cells (Fig. 3A, C a, b). In cells transfected with a plasmid containing HIF-1α cDNA (HIF-1α), HUVEC tube formation was markedly increased (Fig. 3B, D a, b).

### Upregulation of HIF-1α in ADSCs sheets increases osteointegration, bone formation, and osteoid mineralization speed around implants

**Fig. 4:**
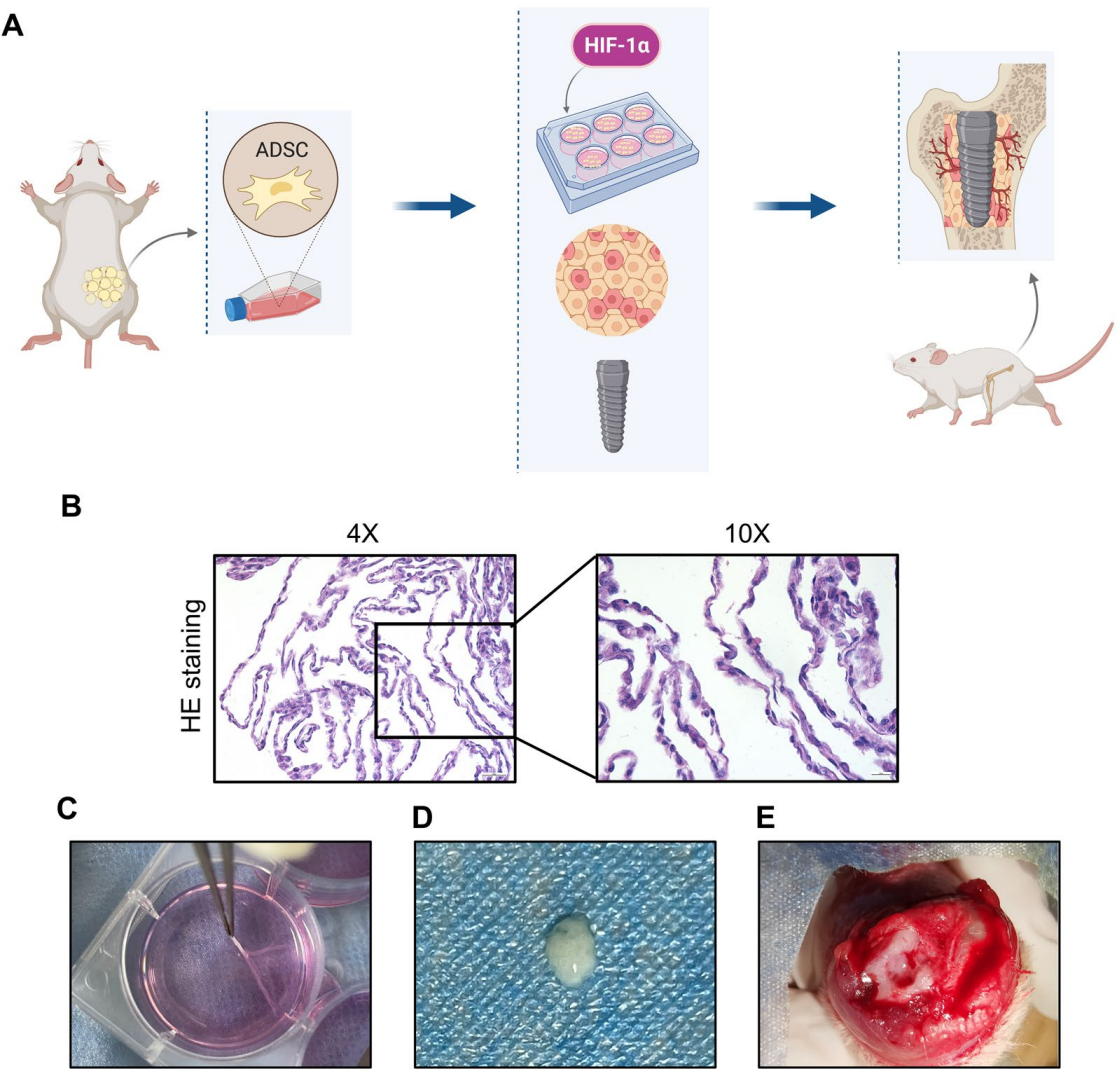
Surgical procedures and treatments. **A** Transfected ADSCs sheet-wrapped titanium implants (length = 5 mm, diameter = 2 mm) were inserted into the femoral condyles of SD rats. Animals were sacrificed after 4 weeks after implantation and the femurs were harvested for radiographic and histological analysis. **B**–**D** HE staining and photographs of ADSC sheets peeled off after 10 days cultured in sheet-forming inducing medium. **E** ADSC sheet was placed and subsequent implant placement

**Fig. 5:**
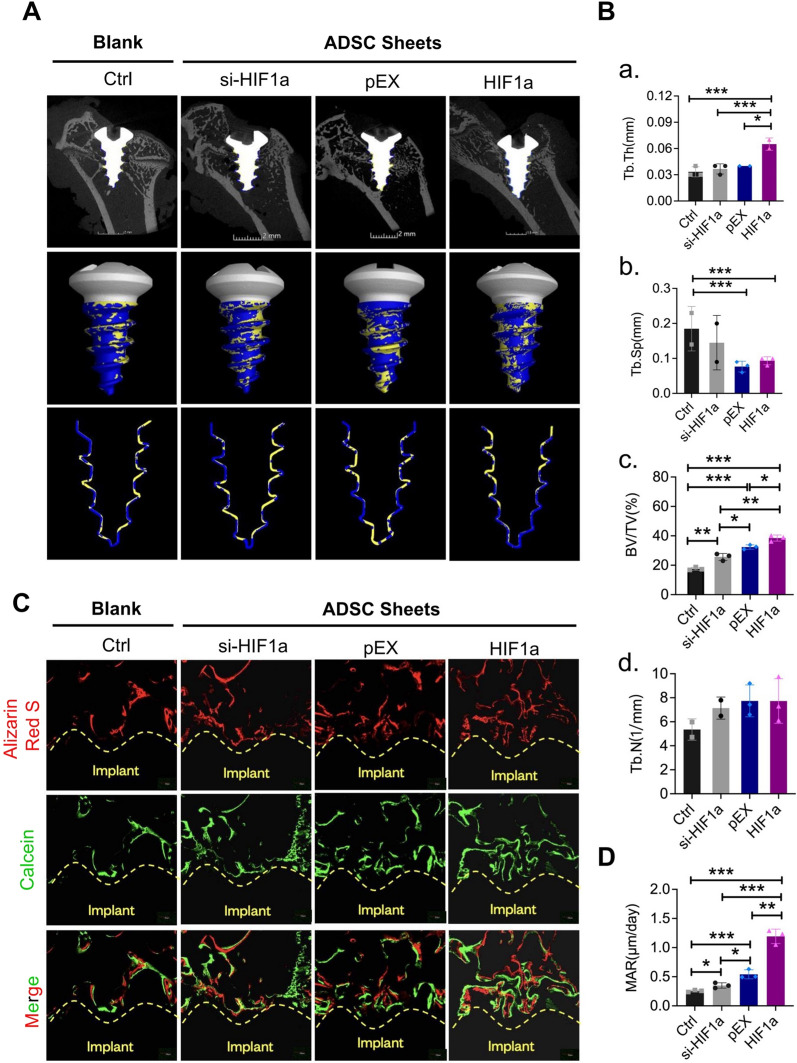
Analysis of bone mass and the microstructure of newly formed bone around implants in vivo. **A** Microcomputed tomography analysis of (**a**) two-dimensional images of rat femoral condyles, (**b**) the reconstructed three-dimensional structures of the region of interest (ROI), including the bone-to-implant contact area (yellow) and noncontact area (blue), (**c**) and a sagittal reconstruction of the ROI with the bone-to-implant contact area (yellow) and non-contacting area (blue). **B** Evaluations of all morphometric parameters within the ROI, including (**a**) Tb. Th, (**b**) Tb.Sp, (**c**) BV/TV, and (**d**) Tb. N in each group, (BV/TV: percentage of the bone volume; Tb. Th: trabecular thickness; Tb. N: trabecular number; Tb.Sp: trabecular separation). **C** New bone formation by double fluorescence labeling method with Alizarin red (red) and Calcein (green) double fluorescence labeling in rat femoral condyles. **D** Quantitative analysis of the speed of osteoid mineralization by MAR. *p < 0.05; **p < 0.01; ***p < *0.001*

To further evaluate the ability of the HIF-1α gene to improve the osteogenic capacity of ADSCs, titanium implants wrapped with sheets of transfected ADSCs (length = 5 mm, diameter = 2 mm) were inserted into the femoral condyles of SD rats (Fig. 4). Four groups were designed, as follows: a blank control group without an ADSCs-sheet, a group with an si-HIF1α-ADSC-sheet; a group with a HIF1α-ADSC-sheet; and a negative control group with a pEX-ADSCs-sheet. Micro-CT scanning and reconstruction was performed to analyze the bone structure around the implants in the region of interest (ROI) (within 40 μm of the implant surface). The bone-implant osseointegrated area is shown in yellow, whereas the non-contacting area is shown in blue (Fig. 5A); analysis of the bone microstructure in the ROI showed higher BV/TV and Tb.Th values and lower Tb.Sp values in the group with implants wrapped in sheets of HIF-1α-ADSCs. The group with implants wrapped in sheets of si-HIF-1α ADSCs exhibited lower BV/TV, Tb.Th values and higher Tb.Sp values, while the values of the negative control group were between those of the other groups. However, the blank control group without an ADSC sheet has the smallest bone-to-implant contact area, Tb.Th value, and Tb.N value but the highest trabecular separation (Fig. 5B a–d).

Sequential fluorescence labeling experiments were performed to observe whether sheets of transfected ADSCs could increase osteoid mineralization speed during the observation period. We intraperitoneally administered Alizarin red (red) and Calcein (green) sequentially at 3 days and 10 days before sacrifice, respectively. In SD rats, there were fewer fluorescence labeled lines showing newly formed bone around the implants in the blank control group than in the groups with implants wrapped in ADSCs sheet, while the group with implants wrapped in sheets of ADSCs subjected to HIF-1α upregulation exhibited significantly increased new bone formation. The mineral apposition rate (MAR) also validated the previous results, with the group with implants wrapped in sheets of HIF-1α-overexpressing ADSCs exhibiting the fastest osteoid mineralization speed. However, downregulation of HIF-1α in the ADSC sheets significantly impaired new bone formation and decreased the mineral apposition rate, as expected (Fig. 5C, D).

### Histological analyses of osseointegration and new bone formation around implants

**Fig. 6:**
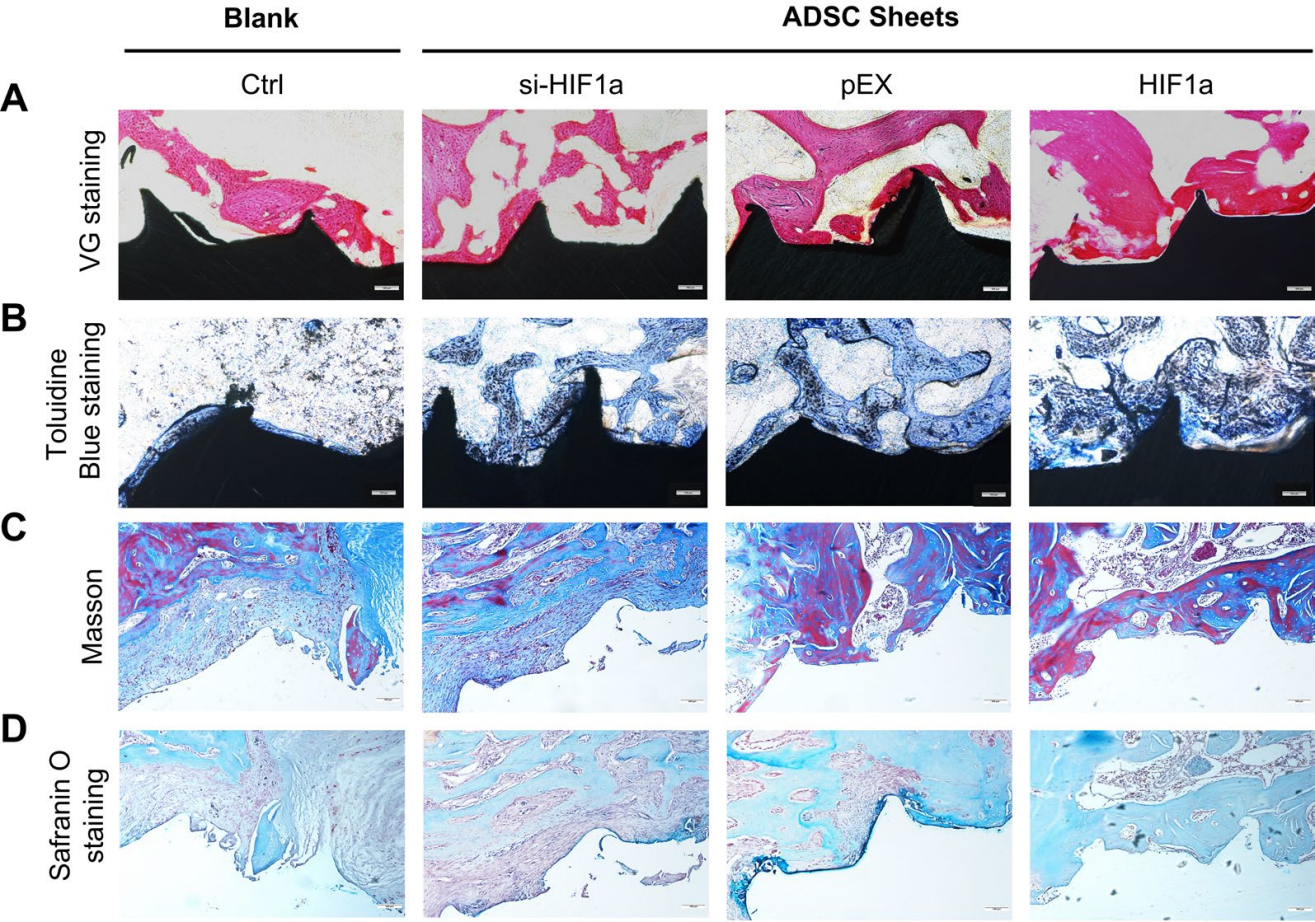
Osteogenesis and new bone formation were observed 30 days after surgery. **A** VG staining, **B** toluidine blue staining, **C** Masson staining, and (**D**) Safranin O staining under light microscopy. Scale bar = 100 μm

To further confirm the osteogenesis-promoting effect of wrapping implants with sheets of ADSCs subjected to HIF-1α upregulation, a series of staining assays were carried out to evaluate osseointegration and new bone formation post-operation. The undecalcified sections were subjected with VG staining (Fig. 6A) and toluidine blue staining (Fig. 6B), and large areas of the bone trabecula structure were in contact with the surface of the implants in the group with implants wrapped in sheets of HIF1α-ADSCs. However, the results showed slight increase in bone mass and osseointegration in the si-HIF1α-ADSCs-sheets and blank groups, with the blank control group showing the worst result. We also performed Masson-trichrome staining and Safranin O staining to visualize new bone formation. After Masson staining, the group with implants wrapped in sheets of ADSCs subjected to HIF1α upregulation showed increased osseointegration, with a large amount of mature bone tissue (stained in red) mixed with a small amount of newly formed bone (stained in blue) in contact with the implant (Fig. 6C). The same trend was observed in Safranin O staining; the HIF1α ADSC sheets group showed increased in bone-implant contact ratio, with a greater proportion of bone formation (staining in green) than cartilage formation (staining in red) (Fig. 6D). In contrast, the si-HIF1α group had the least new bone formation among the three ADSC sheet groups and significantly less bone mass and osseointegration than blank control group.

### Involvement of the HIF-1α/VEGF/AKT/mTOR signaling pathway in osteogenic differentiation in ADSCs

**Fig. 7:**
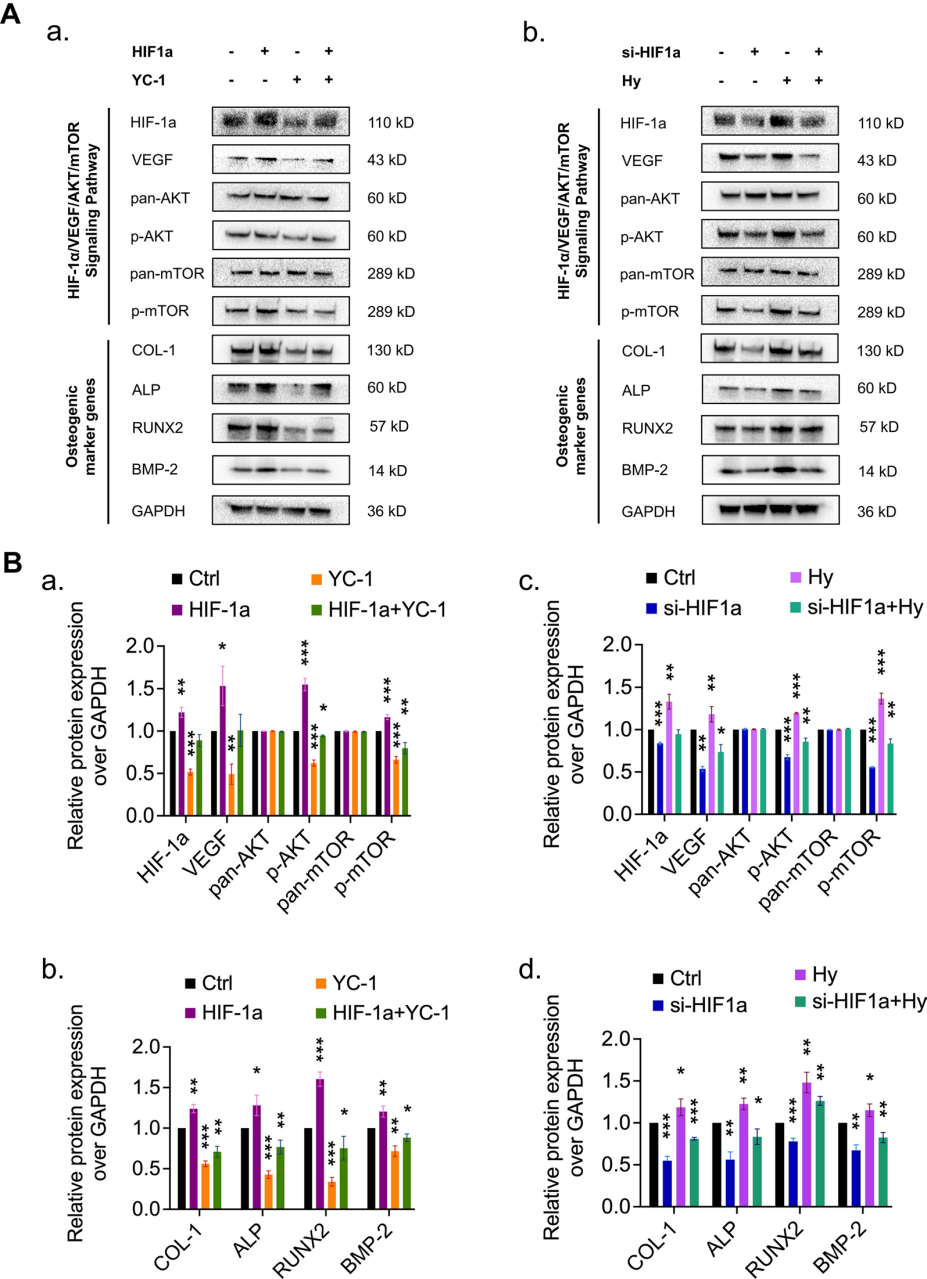
HIF-1α inhibited and promoted ADSCs osteogenic differentiation by regulating VEGF/AKT/mTOR expression. **A**, **B** Western blotting analysis shows that protein expression of HIF-1α and VEGF and the phosphorylation of AKT and mTOR in ADSCs exposed to no treatment, a small interfering RNA targeting HIF-1α (si-HIF1α), and plasmids containing HIF-1α cDNA (HIF1α). Hypoxia (2% O_2_) and 3-(5′-Hydroxymethyl-2′furly)-1-benzy indazole (YC-1, 10 μmol/l) was used to antagonise the effects of si-HIF1α and HIF-1α, respectively

Several studies have reported that the regulatory mechanism of HIF-1α may involve activating of the AKT/mTOR pathway [51]. This led us to ask whether the increases in ADSCs proliferation, migration, and osteogenic differentiation observed after upregulating HIF-1α are related to the VEGF/AKT/mTOR signaling pathway. Thus, we treated ADSCs with small interfering RNA targeting HIF-1α (si-HIF-1α) and a plasmid containing HIF-1α cDNA (HIF-1α). Hypoxia (2% O_2_) and 3-(5′-Hydroxymethyl-2′furly)-1-benzy indazole (YC-1, 10 μmol/l) was used to antagonise the effects of si-HIF1α and HIF-1α, respectively. Then, the protein expressions of HIF-1α, VEGF, pan-AKT, p-AKT, pan-mTOR, p-mTOR, ALP, COL-1, RUNX2, and BMP-2 were evaluated by Western blotting (Fig. 7). Overall, the HIF-1α/VEGF/AKT/mTOR signaling pathway facilitated the proliferation, focal adhesion, migration, and osteogenic differentiation in ADSCs.

## Discussion

In this study, we showed that ADSC sheets promoted osseointegration of titanium implants in rats. Furthermore, the knock down of HIF-1α in ADSC sheets wrapped around the implants eliminated peri-implant osseointegration, whereas upregulation of HIF-1α in ADSC sheets wrapped around the implants promoted osseointegration and bone formation. Our observations suggest a potential novel clinical strategy using ADSCs modified to overexpress of HIF-1α in bone defect and repair.

Considering the osteogenic potential of ADSCs in bone formation and repair under hypoxic conditions, we hypothesized that HIF-1α might play a vital role in peri-implant osteogenesis, and we conducted a series of in vitro and in vivo experiments to investigate different expression levels of HIF-1α. Silencing HIF-1α obviously hampered the proliferation and migration of ADSCs, resulting in a significant increase in scratch area in the scratch wound healing assay and a marked decrease in the number of migrated cells in the Transwell migration assay. Furthermore, compared to the control treatment, knocking down HIF-1α inhibited the osteogenic ability of ADSCs after 7 days and 21 days of osteogenic induction, which was demonstrated by significant decreases in ALP activity and the number of calcium nodules in ADSCs. This phenomenon was also confirmed by downregulation of the mRNA and protein levels of bone formation- related factors such as ALP, RUNX2, COL-1 and BMP-2. However, when HIF-1α was overexpressed, we observed the opposite phenomenon, as the proliferation, migration, and osteogenic ability of ADSCs were enhanced. In addition, overexpression of HIF-1α significantly upregulated the expression of osteogenesis-related mRNAs and proteins such as ALP, RUNX2, COL-1 and BMP2, in ADSCs [52, 53]. These results were consistent with our other findings and suggested that HIF-1α plays a key role in osteogenesis in ADSCs.

To better support the application of these research results in clinical practice, we carried out in vivo experiments involving tissue engineering combined with gene mutation of HIF-1α. We modified ADSC cell sheets via HIF-1α overexpression or knockdown, and titanium implants wrapped with the transfected ADSC sheets were then inserted into the femoral condyles of SD rats. We observed that the HIF-1α-modified ADSC cell sheet could significantly promote the peri-implant osteointegration. Bone formation parameters, such as BV/TV, Tb.Th, Tb.Sp and TB.N were evaluated by micro-CT scanning of the implants and the surrounding bone tissue. The BV/TV and Tb.Th values in the HIF-1α-modified cell sheet group were significantly higher than those in the control group, and the Tb.Sp value was significantly lower in the HIF-1α-modified cell sheet group than in the control group. This indicated that the peri-implant bone mass, thickness of new bone, and velocity of bone formation were significantly increased after HIF-1α overexpression. However, ADSC cell sheets modified by knockdown of HIF-1α significantly decreased the peri-implant bone mass, thickness of new bone, and rate of new bone formation. This conclusion was further confirmed by double fluorescence labeling with Alizarin red stain (red) and calcein (green). In the HIF-1α overexpression group, the amount and the speed of bone formation were both better than those in the control group. Moreover, the histological sections stained with VG, toluidine blue, Masson, and safranin O also supported this conclusion.

It has been widely reported that the VEGF/AKT/mTOR signaling pathway plays key roles in cell proliferation, migration, angiogenesis, and osteogenesis in anoxic environment [54, 55]. In the present study, we confirmed the role of this important signaling pathway and observed that overexpression of HIF-1α could upregulate VEGF expression at the protein level in ADSCs, thus activating the VEGF/AKT/mTOR signaling pathway and leading to increased p-AKT and p-mTOR levels. The upregulation of VEGF occurred because HIF-1α, as a transcription factor of VEGF, could recognize HRE enhancers and initiate the transcription of VEGF [56]. The upregulation of VEGF could promote angiogenesis, thus promoting bone formation around implants [57]. On the other hand, VEGF can bind to VEGF receptor (VEGFR) to phosphorylate AKT, thus increasing p-AKT levels [58]. The data also showed that HIF-1α activated AKT and mTOR but did not increase the expression of total-AKT and total mTOR. Moreover, overexpression of HIF-1α blocked the downregulation of p-AKT and p-mTOR induced by YC-1, an antagonist of HIF-1α [59]. This was consistent with previous research showing that the upregulation of p-AKT protein expression by HIF-1α was partially achieved via upregulation of VEGF. Therefore, these results suggest that HIF-1α is a pivotal transcriptional regulator of cell biological functions and affects osteogenic differentiation via the HIF-1α/VEGF/AKT/mTOR signaling pathway.

However, there are still some limitations of this study. For example, ADSC cell sheets are soft, and the thickness is uneven when they are wrapped around the implant; in addition, the ADSC sheet may be damaged or even broken during the implantation process. This could affect peri-implant osteointegration and primary implant stability. Therefore, modifications that enabling the cell sheets to play a more consistent biological role are worth considering. It was reported that Gelatin meth acryloyl hydrogels could simulate the 3D microenvironment in vivo [60]. Furthermore, hydrogels loaded with stem cells could greatly accelerate stem cell differentiation and bone formation [61], suggesting that incorporating ADSCs into the hydrogel system could optimize the role of ADSC sheet. Future studies should be performed to test this hypothesis.

## Conclusion

Overall, this study revealed that ADSC sheets modified by the upregulation of HIF-1α promoted osteointegration of titanium implants by coupling angiogenesis and osteogenesis via the HIF-1α/VEGF/AKT/mTOR signaling pathway. This provides an important theoretical basis for improving the clinical application of ADSCs to promote peri-implant osteointegration.

## Methods

### Animals and ethical considerations

All animal procedures were performed in accordance with the guidelines of the Institutional Animal Care and the Use Committee of China. The experiments were approved by the Ethics Committee of the School of Stomatology, Fourth Military Medical University (Appl. No. k9-2022-004). A total of 30 eight- to nine-week-old male rats (Chengdu Dossy Experimental Animals Co., Ltd., Chengdu, China) were acquired for ADSC isolation and animal experiments. All SD rats were maintained under pathogen-free conditions with a temperature of 25 °C, 55% humidity, and 12 h of light alternating with 12 h of darkness.

### Isolation, culture, and identification of rat ADSCs

ADSCs were surgically isolated from subcutaneous adipose tissue from the inguinal area and digested with an equal volume of 0.2% type I collagenase at 37 °C for 60 min. After filtering with a 200-mesh and centrifugation at 1000×*g* for 5 min, the supernatant was removed, and the precipitate was resuspended in complete alpha-MEM (10% fetal bovine serum from Gibco, USA, and 1% penicillin/streptomycin from HyClone, USA) and cultured in T75 culture flasks in an incubator (37 °C, 5% CO_2_). The culture medium was changed every 48 h, and the cells were passaged after reaching 80% confluence. The cells used in the following experiments were from passage 3. Images of cell morphology were captured under an inverted phase-contrast microscope. To evaluate the characteristics of ADSCs, the cell surface markers CD29, CD44, CD45, and CD105 were investigated by flow cytometry.

### HIF-1α siRNA and cDNA transfection

Small interfering RNA (siRNA) transfection was performed for HIF-1α gene silencing using siRNA from GenePharma, with the sequence GGGCCGUUCAAUUUAUGAATT (si-HIF-1α). ADSCSs were cultured on 6-well plates in complete alpha-MEM until they reached 60–70% confluence. Lipofectamine 3000 (Invitrogen, Waltham, MA, USA) was used according to the manufacturer’s protocol to transfect HIF-1α siRNA Silencer (si-HIF-1α) or nontargeting siRNA as a negative control (si-NC). ADSCs were cultured and maintained at hypoxic culture conditions at 2% O_2_, 5% CO2, 37 ºC in humidified incubators. Total RNA and protein were collected after 24 h of cultivation. Plasmid containing HIF-1α cDNA (HIF-1α) or empty plasmid (pEX) was transfected into ADSCs using Lipofectamine 3000 (Invitrogen, Waltham, MA) according to the manufacturer’s instructions. After 24 h of transfection, the cells were washed with PBS, and the medium was replaced with complete alpha medium for the remaining experiments. Total RNA was extracted after 48 h of transfection for qRT‒PCR assays to detect the transfection efficiency of HIF-1α in ADSCs.

### Cell proliferation assays

ADSCs were seeded into 96-well culture plates at a density of 3 × 10^3^ cells per well and given different treatments according to the experimental purpose. Cell-counting kit-8 (CCK-8; 10 μl per well; PCM, Xi’an, China) solution was used to assay the proliferation after 0, 24, 48, and 72 h. Three repeated experiments were conducted, and the optical density of the medium was detected at 450 nm using a multifunction enzyme labeling instrument (Thermo Fisher Scientific, Waltham, MA, USA).

### Scratch wound healing assay and Transwell assay

The migration ability of ADSCs was measured using scratch wound healing assays and Transwell assays (8.0 μm pore size; Corning-Costar). For the scratch assay, 1 × 10^6^ ADSCs per well were seeded into 6-well culture plates and given various treatments according to the experimental design. After the cells reached 100% confluence, a 200 μl pipette tip was used to scratch the monolayer, and the well was washed with PBS to rinse off floating cells. The cells were then cultured in serum-free alpha-MEM and photographed after 0, 12, and 24 h. For the Transwell migration assay, a total of 2 × 10^5^ ADSCs in 200 μl serum-free alpha-MEM were added to the upper chamber, and 800 μl complete alpha-MEM was added to the bottom chamber. After 24 h of cultivation, the migrated cells on the bottom chamber were removed, fixed using 4% PFA for 15 min and stained with 0.5% crystal violet for further evaluation.

### Alkaline phosphatase (ALP) and Alizarin red staining (ARS)

ALP activity was assayed using a BCIP/NBT alkaline phosphatase color development kit (Beyotime Biotech, Shanghai, China) and an alkaline phosphatase (ALP) assay kit (Jiancheng, Nanjing, China) was purchased for quantification of ALP activity following the manufacturer’s protocol. Calcium deposits were detected by staining with 2% alizarin red S (Solarbio, Beijing, China). To quantify the stained nodules, solubilized stain was transferred to the wells of a 96-well plate and measured at 620 nm of absorbance. All data are presented as the means (n = 3).

### ADSC adhesion test and immunofluorescence staining

Prior to the cell adhesion test, prepared titanium sheets with a diameter of 12 mm and a thickness of 2 mm (n = 3 in each group) were immersed in 5 ml of 95% alcohol for 48 h and subjected to ultraviolet irradiation for complete disinfection. Transfected ADSCs (3 × 10^3^/well) were centrifuged and seeded into 24-well culture plates with titanium sheets already placed in the wells. After 24 h, the culture medium was removed, and the titanium sheet samples were washed with a PBS buffer 3 times. The samples were fixed in 4% paraformaldehyde for 10 min at room temperature, permeabilized, and visualized using double fluorescence staining for the cell cytoskeleton (Alexa Fluro 635 phalloidin dye, Invitrogen) in green and nuclei (DAPI dye) in blue. The number of adherent cells and the morphology and spreading of the ADSCs were observed by confocal laser scanning microscopy (OLYMPUS, Tokyo, Japan).

### Tube formation assay

HUVECs were grown and transfected with a small interfering RNA targeting HIF-1α (si-HIF-1α) and a plasmid containing HIF-1α cDNA (HIF-1α) for 24 h, and then HUVECs (3 × 10^4^ cells per well) were seeded on 96-well plates. Each well was coated with 200 μl Matrigel (BD Biosciences, San Jose, CA, USA), and the HUVECs were cultured for 18 h at 37 °C in 5% CO_2_. Evident capillary-like structures were counted using a phase-contrast microscope, and the networks formed by HUVECs were quantified with VIDEOMET software (Videojet Technologies Inc., Chicago, IL, USA). Quantitation of angiogenic activity during tube formation was performed by counting the number of junctions and total branch points. Three independent assays were performed. Data are summarized as the means ± SDs.

### Quantitative real-time qPCR

To determine the relative mRNA expression levels of HIF-1α, ALP, RUNX2, type I collagen (COL-I), and BMP2 in ADSCs, 1 × 10^6^ ADSCs per well were seeded into 6-well culture plates and incubated with various treatments for 24 h prior to total RNA extraction. cDNA was reverse transcribed from the extracted RNA (Applied Biosystems, Foster City, CA, USA) using SYBR Premix ExTaq II (TaKaRa, Tokyo, Japan) along with a StepOne Plus Real-Time PCR system (used to perform qPCR analysis). The fold change in the mRNA expression levels of each target mRNA was calculated using the 2^−△△CT^ method and normalized to the internal reference glyceraldehyde 3-phosphate dehydrogenase (GAPDH). The sequences of all primers (Sangon Biotech, Shanghai, China) used in the present study are shown in Additional file 1: Table S1.

### Protein extraction and Western blotting

Total proteins were lysed and extracted using RIPA buffer (ZHHC, China) after seeding (1 × 10^6^ cells/well, 6-well culture plates) and incubated with various treatments for 24 h. A BCA Protein Assay Kit (Beyotime Biotechnology, China) was used to quantify the protein concentration. The following primary antibodies used for this study were obtained from Abcam Biotechnology (MA, USA): anti-HIF-1α (1:2000, ab179483), anti-GAPDH (1:10,000, ab181602), anti-ALP (1:2000, ab307726), anti-RUNX2 (1:2000, ab236639), anti-type I collagen (COL-I) (1:2000, ab260043), anti-BMP2 (1:2000, ab284387), anti-p-AKT (1:2000, ab81283), anti-AKT (1:2000, ab8805), anti-mTOR (1:2000, ab134903), and anti-p-mTOR (1:2000, ab109268). The membrane was incubated at 4 °C and then rinsed three times in TBS-T (5 min/wash) at RT. After 1 h of incubation with a secondary antibody (Boster, Wuhan, China) at RT, followed by rinsing with TBS-T, visualization was performed with a chemiluminescence imaging system (Bio-Rad GelDoc XR + , Hercules, CA, USA).

### Fabrication of ADSC sheets

Third generation ADSCs were seeded at 1 × 10^6^ cells/well in a plate containing 6 wells. After reaching approximately 90% confluence, sheet formation-inducing medium was used instead of basic medium. The composition of the culture medium was α-MEM (Gibco, USA) with 10% bovine fetal serum (Sijiqing, China), 1% penicillin/streptomycin (HyClone, USA), and 50 mg/ml vitamin C (NCM, China). ADSCs were cultured in sheet formation-inducing medium for 10 days, and the nutrient solution was replaced every 3 days. When a curly edge appeared at the plate rim, the whole cell sheet was peeled off with a scraper [62].

### Surgical procedure and treatment

The experiments were approved by the Ethics Committee of the School of Stomatology, Fourth Military Medical University (Appl. No. k9-2022-004). The guidelines of the Institutional Animal Care and Use Committee of China were followed. The experiment consisted of four groups (n = 5 in each group): (1) the blank control group: rats subjected to only implant placement, (2) the si-HIF-1α ADSC sheet-wrapped group: SD rats with sheets of ADSCs transfected with a HIF-1α silencing construct wrapped around the placed implants, (3) the pEX ADSC sheet-wrapped group: SD rats with sheets of ADSCs transfected with nontargeting siRNA wrapped around the placed implants, and (4) the HIF-1α ADSC sheet-wrapped group: SD rats with sheets of ADSCs transfected with a HIF-1α overexpression construct wrapped around the placed implants. The implants used in this study were from Kontour Medica (Xi’an, China) and had a length of 5 mm and a diameter of 2 mm. The rats were subjected to general anesthesia with 1% pentobarbital solution (45 mg/kg rat weight). The animals were placed on a heating pad to maintain body temperature. After shaving the hind limb, the femoral condyles were exposed by a longitudinal incision on the lateral side of the knee joint. A hole was created in the femoral condyles parallel to the long axis of the femora, and the implants were first wrapped with transfected ADSC sheets and then placed into the holes. The incisions were sutured in layers carefully. On the tenth and third days before sacrifice, alizarin red S (30 mg/kg) and calcein (20 mg/kg) (Sigma‒Aldrich, St, Louis, MO, USA) were administered sequentially via the intraperitoneal route. Animals were sacrificed 4 weeks after implantation, and the femurs were harvested for radiographic and histological analysis.

### Micro-CT analysis

The specimens were fixed overnight in 70% ethanol (n = 3 in each group) and subjected to microcomputed tomography scanning (Siemens Inveon, Erlangen, Germany) to identify alterations in the peri-implant tissue. The region of interest (ROI) within 40 μm of the implant surface was subjected to three-dimensional reconstruction to analyze the morphometric parameters of the bone around implants, including the bone volume percentage (BV/TV, %), trabecular thickness (Tb.Th, mm), and trabecular number (Tb.N, 1/mm), and trabecular separation (Tb.Sp, mm).

### Sequential fluorescence labeling and histological analysis

The specimens were dehydrated through a gradient ethanol series (75–100%), infiltrated in methyl methacrylate, and then embedded in poly-methyl-methacrylate resin (n = 3 in each group). A hard tissue slicer (Leica SP1600, Nussloch, Germany) was used to cut each specimen through the center of the implant parallel to its long axis, and each section was approximately 300 μm in thickness. The slices were then ground and polished to a sheet of 80 μm and directly observed by confocal laser scanning microscopy (OLYMPUS, Tokyo, Japan) with spectral excitation at different wavelengths. To quantify the speed of osteoid mineralization during the observation period in the four groups, the mineral apposition rate (MAR) was measured.

### Histological evaluation

Methylene blue acid fuchsin staining (VG staining), toluidine blue staining, Masson trichrome staining, and Safranin O staining were performed on undecalcified sections to visualize new bone formation (n = 3 in each group).

### Statistical analysis

Quantitative data are described as the means ± SDs of at least three independent experiments, and statistical analyses were performed using GraphPad Prism 5.0 (GraphPad Software, San Diego, CA, USA). Images were analyzed using ImageJ and Image Pro. Quantitative data across all groups were analyzed using one-way analysis of variance (ANOVA) and *t tests*. A P value less than 0.05 was considered to indicate statistical significance. The comparison results were labeled with * for P value < 0.05; ** for P value < 0.01 and *** for P value < 0.001.

### Supplementary Information

**Additional file 1: Figure S1. **Characterization of ADSCs and detection of ADSC differentiation. (A) Representative image of ADSCs at passage 3. (B) Representative image of the osteogenesis of ADSC osteogenesis. (C) Fat droplets stained as a marker of adipogenesis in ADSCs. (D) Flow cytometry histograms of ADSCs exhibiting clear expression of CD29, CD44 and CD105 and no expression of CD45. Scale bar = 200μm). **Figure S2. **Preliminary experiment of the (A). HIF-1a protein expression levels in ADSCs under hypoxia conditions. (B). Quantitative analysis of the HIF-1a protein expression levels. **Table S1. **Primer sequences used in real-time PCR

## Data Availability

All data generated or analyzed during this study are included in this published article.

## Abbreviations

ADSCs: Adipose-derived stem cells
BMSCs: Bone marrow mesenchymal stem cells
BMMs: Bone marrow-derived macrophages
HUVECs: Human umbilical vein endothelial cells
SD rats: Sprague–Dawley rats
siRNA: Small interfering RNA
BV/TV: Bone volume percentage
Tb.Th: Trabecular thickness
Tb.N: Trabecular number
Tb.Sp: Trabecular separation
α-MEM: α-Minimum essential medium
HIF-1α: Hypoxia-inducible factor 1α
AKT: Phosphorylation protein kinase B
ALP: Alkaline phosphatase
p-mTOR: Phosphorylated mammalian target of rapamycin
RUNX2: Runt-related transcription factor
Col1: Type 1 collagen
BMP2: Bone morphogenetic protein 2
VEGF: Vascular endothelial growth factor
PGF: Placental growth factor
