# Supplementary material for: HIF-1α increases the osteogenic capacity of ADSCs by coupling angiogenesis and osteogenesis via the HIF-1α/VEGF/AKT/mTOR signaling pathway
Source: J Nanobiotechnology. 2023 Aug 7;21:257. doi: 10.1186/s12951-023-02020-z (PMC10405507; doi:10.1186/s12951-023-02020-z)
Supplement: Supplementary file 1 — Additional file 1: Figure S1. Characterization of ADSCs and detection of ADSC differentiation. (A) Representative image of ADSCs at passage 3. (B) Representative image of the osteogenesis of ADSC osteogenesis. (C) Fat droplets stained as a marker of adipogenesis in ADSCs. (D) Flow cytometry histograms of ADSCs exhibiting clear expression of CD29, CD44 and CD105 and no expression of CD45. Scale bar = 200μm). Figure S2. Preliminary experiment of the (A). HIF-1a protein expression levels in ADSCs under hypoxia conditions. (B). Quantitative analysis of the HIF-1a protein expression levels. Table S1. Primer sequences used in real-time PCR [file 12951_2023_2020_MOESM1_ESM.docx]

**
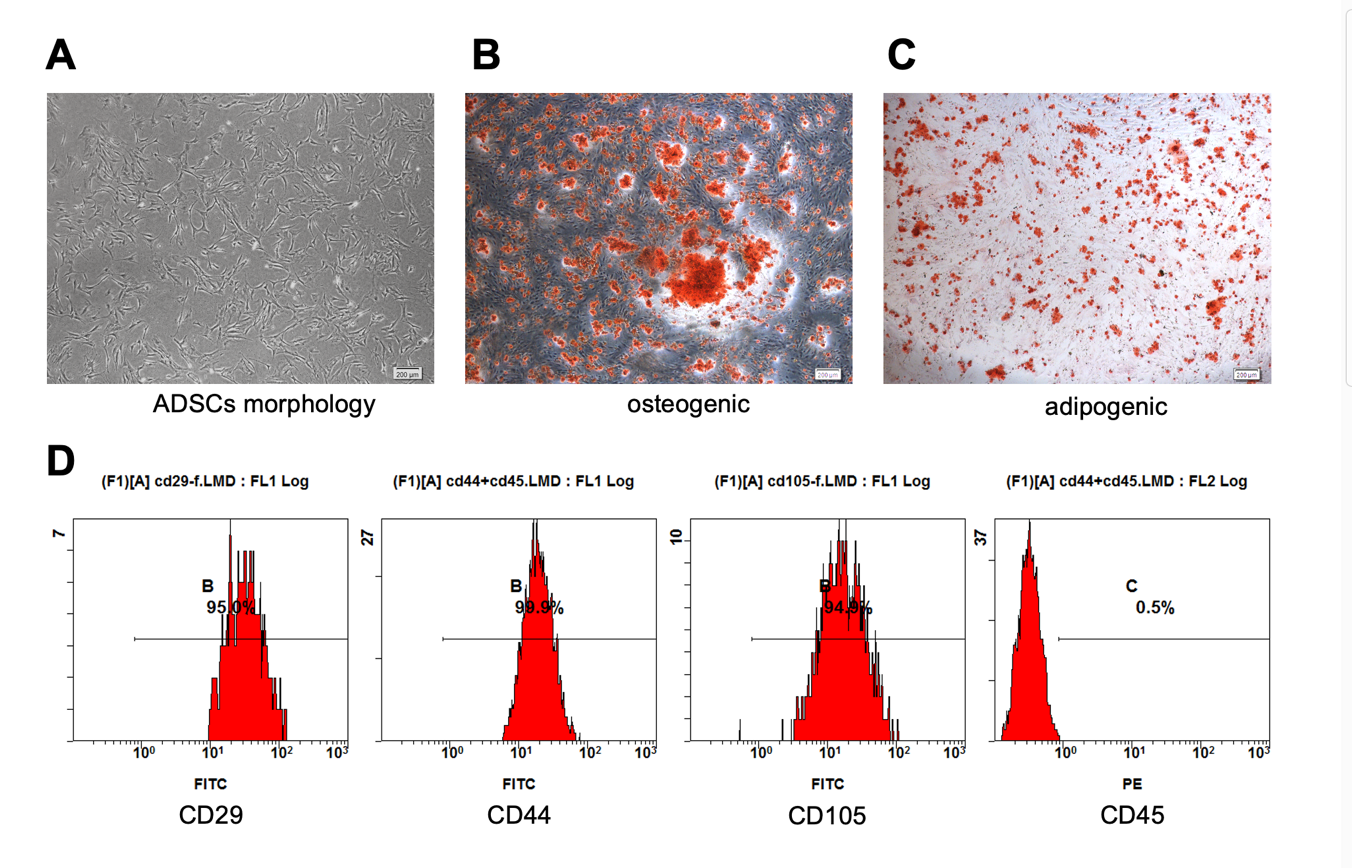
**

**Fig. S1.** Characterization of ADSCs and detection of ADSC differentiation. (A) Representative image of ADSCs at passage 3. (B) Representative image of the osteogenesis of ADSC osteogenesis. (C) Fat droplets stained as a marker of adipogenesis in ADSCs. (D) Flow cytometry histograms of ADSCs exhibiting clear expression of CD29, CD44 and CD105 and no expression of CD45. Scale bar = 200μm).


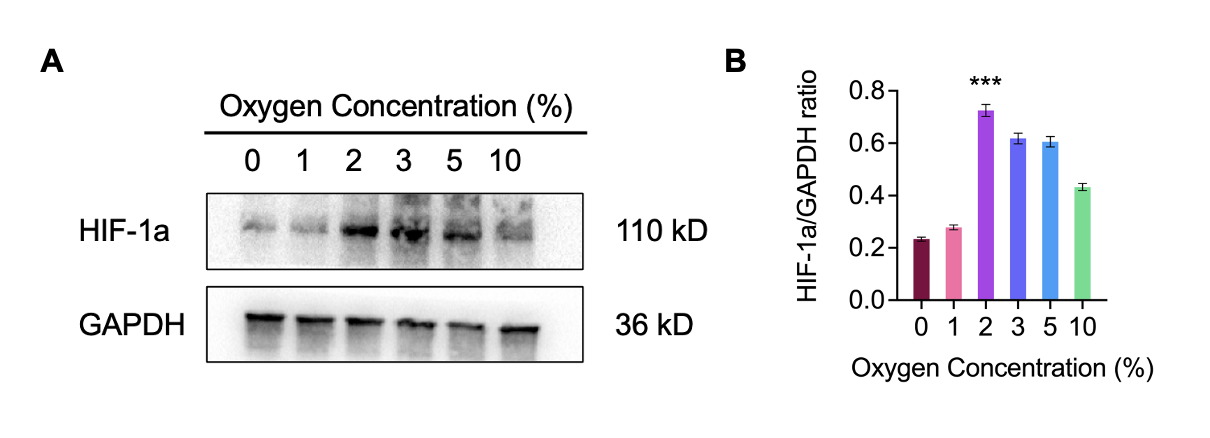


**Fig. S2.** Preliminary experiment of the (A). HIF-1a protein expression levels in ADSCs under hypoxia conditions. (B). Quantitative analysis of the HIF-1a protein expression levels.

**
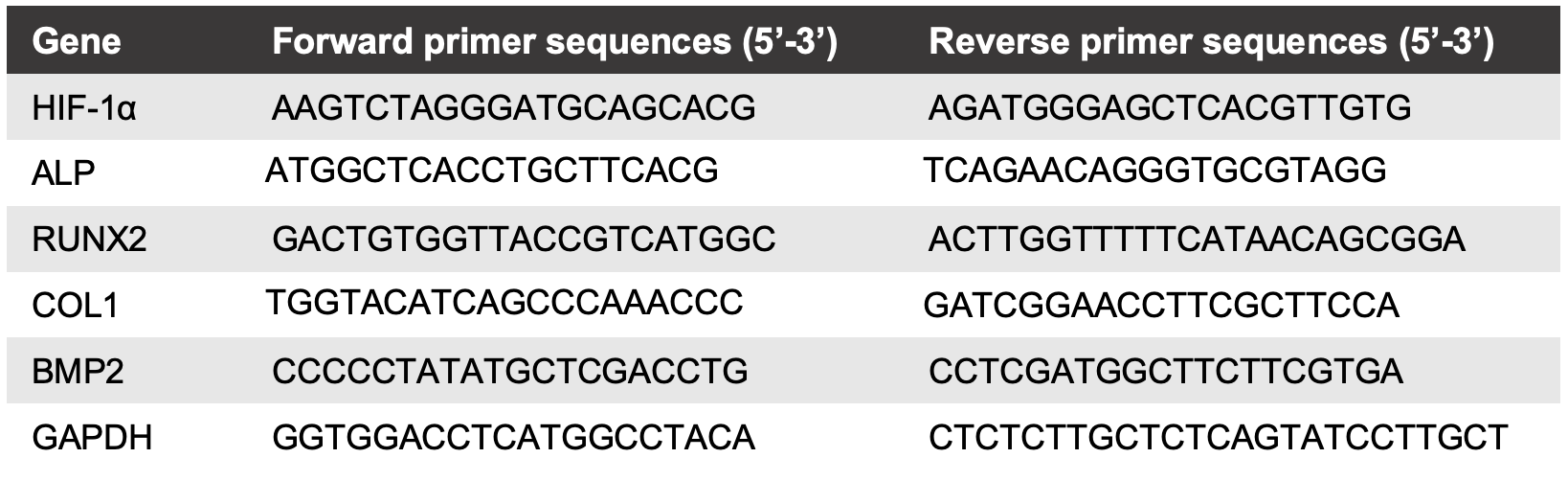
**

**Table S1.** Primer sequences used in real-time PCR
